# Botulinum Neurotoxin Serotype A Recognizes Its Protein Receptor SV2 by a Different Mechanism than Botulinum Neurotoxin B Synaptotagmin

**DOI:** 10.3390/toxins8050154

**Published:** 2016-05-17

**Authors:** Jasmin Weisemann, Daniel Stern, Stefan Mahrhold, Brigitte G. Dorner, Andreas Rummel

**Affiliations:** 1 Institut für Toxikologie, Medizinische Hochschule Hannover, Carl-Neuberg-Str. 1, 30625 Hannover, Germany; weisemann.jasmin@mh-hannover.de (J.W.); mahrhold.stefan@mh-hannover.de (S.M.); 2 Biological Toxins, Centre for Biological Threats and Special Pathogens, Robert Koch Institute, Seestr. 10, 13353 Berlin, Germany; sternd@rki.de (D.S.); dornerb@rki.de (B.G.D.)

**Keywords:** botulinum neurotoxin, rat and human synaptic vesicle glycoprotein 2, gangliosides, binding constant, surface plasmon resonance

## Abstract

Botulinum neurotoxins (BoNTs) exhibit extraordinary potency due to their exquisite neurospecificity, which is achieved by dual binding to complex polysialo-gangliosides and synaptic vesicle proteins. The luminal domain 4 (LD4) of the three synaptic vesicle glycoprotein 2 isoforms, SV2A‐C, identified as protein receptors for the most relevant serotype BoNT/A, binds within the 50 kDa cell binding domain H_C_ of BoNT/A. Here, we deciphered the BoNT/A‐SV2 interactions in more detail. In pull down assays, the binding of H_C_A to SV2-LD4 isoforms decreases from SV2C >> SV2A > SV2B. A binding constant of 200 nM was determined for BoNT/A to rat SV2C-LD4 in GST pull down assay. A similar binding constant was determined by surface plasmon resonance for H_C_A to rat SV2C and to human SV2C, the latter being slightly lower due to the substitution L563F in LD4. At pH 5, as measured in acidic synaptic vesicles, the binding constant of H_C_A to hSV2C is increased more than 10-fold. Circular dichroism spectroscopy reveals that the quadrilateral helix of SV2C-LD4 already exists in solution prior to BoNT/A binding. Hence, the BoNT/A‐SV2C interaction is of different nature compared to BoNT/B‐Syt-II. In particular, the preexistence of the quadrilateral β-sheet helix of SV2 and its pH-dependent binding to BoNT/A via backbone–backbone interactions constitute major differences. Knowledge of the molecular details of BoNT/A‐SV2 interactions drives the development of high affinity peptides to counteract BoNT/A intoxications or to capture functional BoNT/A variants in innovative detection systems for botulism diagnostic.

## 1. Introduction

Botulinum neurotoxins (BoNTs) produced by the bacteria *Clostridium botulinum* are the most poisonous protein toxins known [1]. They cause the disease botulism due to blockade of acetylcholine release at the neuromuscular junction. Because of their muscle-relaxing properties BoNTs are also extensively used in medical and cosmetic applications [2]. The seven serologically different BoNT serotypes, A to G, comprise four domains, which play individual roles in the intoxication mechanism [3,4]. BoNTs are 150 kDa AB-protein toxins that are composed of a 50 kDa light chain (LC) linked by a disulfide bridge to the heavy chain (HC), the latter being divided into three independent domains: H_N_ (50 kDa translocation domain, *N*-terminus of HC), H_CN_ and H_CC_ (25 kDa each). The latter two domains constitute the H_C_-fragment and are responsible for neuronal binding [5].

Here, the initial low affinity binding to complex polysialo-gangliosides and subsequent high affinity binding to different synaptic vesicle protein receptor takes place [5]. After the receptor-mediated endocytosis the synaptic vesicle (SV) acidifies inducing the BoNT to change its conformation and presumably inserting a pore or channel into the vesicle membrane. The LC, a Zn^2+^-metalloprotease, is translocated through the pore formed by the H_N_ domain into the cytosol of the cell and is released after reduction of the disulfide bridge connecting LC and HC [6]. Here, the hydrolysis of a specific soluble *N*-ethylmaleimide-sensitive factor attachment protein receptor (SNARE) protein takes place; in the case of BoNT/A, synaptosome-associated protein of 25 kDa (SNAP-25) is specifically cleaved at Q197-R198 [7,8]. This cleavage results in inhibition of the neurotransmitter release into the synaptic cleft leading to flaccid paralysis, and eventually to death by respiratory failure.

BoNT/A, B, E, F and G all possess a single ganglioside binding site (GBS) comprising the conserved E(Q)…H(K)…SxWY…G motif [9,10,11], which specifically recognizes the GalNAc3–Gal4 moiety of complex polysialo-gangliosides albeit with submicromolar affinity [12,13,14]. In addition, synaptotagmin-I and -II (Syt-I/-II) serve as protein receptors for BoNT/B, G and the mosaic BoNT/DC [15,16,17,18,19,20,21], whereas the three isoforms of the synaptic vesicle glycoprotein 2 (SV2A-C) were identified as protein receptors for BoNT/A [22,23]. Furthermore, glycosylated SV2A and B exclusively serve as protein receptors for BoNT/E [24]. All three SV2 isoforms were also reported to be co-precipitated by BoNT/D and F from synaptic vesicle lysates, but a direct protein–protein interaction has not been demonstrated so far [10,25,26].

**Figure 1:**
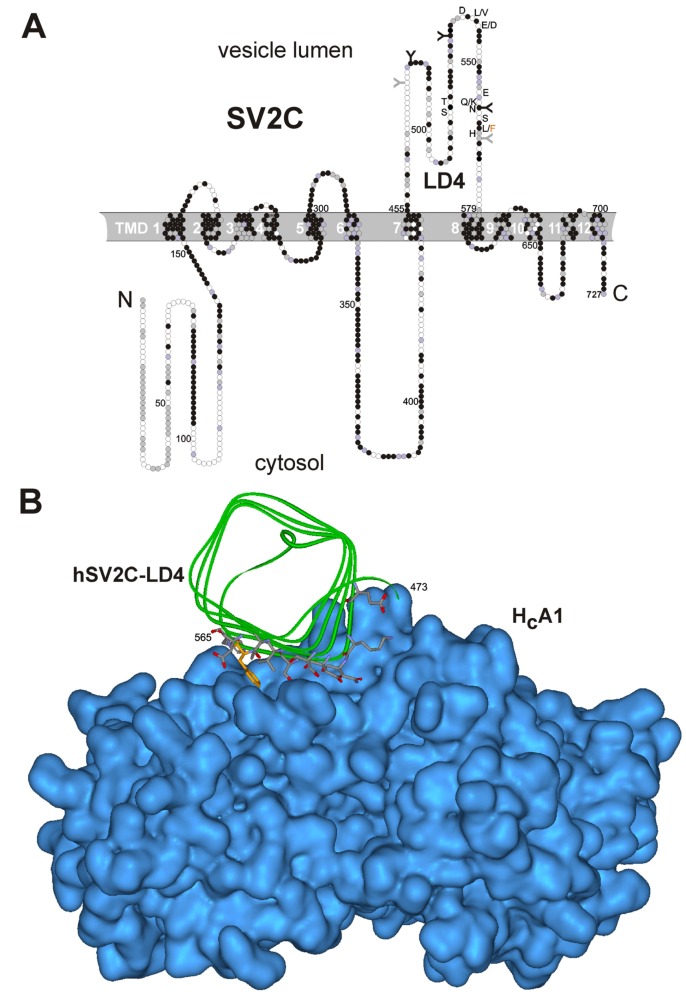
(**A**) Membrane topology of rat synaptic vesicle glycoprotein 2 isoform C (SV2C). Residues identical in rSV2A-C are colored in black, those only conserved in rSV2A or rSV2B in grey. The five putative *N*‑glycosylation sites in SV2C-LD4 (amino acid (AA) 454–579) are depicted as “Y”, those three strictly conserved among SV2A-C in black. The ten residues displaying side chain interactions between human (h) SV2C and H_C_A are indicated in single letter code, the four AA differing to rSV2C are listed additionally. F563 of hSV2C reported to mediate an important cation–π interaction with R1156 of BoNT/A [29] is highlighted in orange (**B**) hSV2C 456–574 (visible only AA 473–565) bound to BoNT/A H_C_ (4JRA.pdb). Surface of H_C_A is shown in blue, the peptide backbone of hSV2C as green ribbon. The ten hSV2C residues displaying side chain interactions with H_C_A are depicted as grey sticks, of them F563 is highlighted in orange.

The three identified highly homologous isoforms SV2A, B and C comprise twelve putative, conserved transmembrane domains (TMD; Figure 1A), are localized in synaptic vesicles and neuroendocrine secretory granules of vertebrates [27], but are differently expressed in the brain [28]. Only the luminal domain 4 (LD4) connecting TMD 7 and 8 of all three SV2 isoforms interacts with BoNT/A H_C_ [22,23]. The human (h) SV2C-LD4 folds into a right-handed, quadrilateral β-helix (Figure 1B) [29]. The corresponding binding site of SV2C within BoNT/A was allocated to the interface of H_CN_ and H_CC_ [29,30] while the Syt binding site locates at the tip of the BoNT/B, G and DC H_CC_ domains in close proximity to the single GBS [11,31,32,33]. In contrast, the SV2C-binding site is found almost opposite to the GBS with a distance of approximately 40 Å. hSV2C binds BoNT/A mainly through backbone-to-backbone interactions at open β-strand edges. In BoNT/A, the amino acids T1145/46, R1156, G1292 and R1294 play an important role in this interaction [29,30]. The co-crystal structure reveals that F563 of hSV2C mediates an important cation–π-stacking interaction with R1156 of BoNT/A (Figure 1B) [29], which, however, is unique to human, chimpanzee and chicken SV2C (Y563 in dog and zebra fish SV2C; H563 in puffer fish; L563 in mouse, rat, bovine; I563 in elephant; M563 in platypus; A563 in camel; T563 in frog; S563 in rabbit; K563 in turtle; D563 in alligator) [34]. The homologous residue 577 of SV2A and 519 of SV2B is a leucine conserved in human, rat, mouse, porcine, dog and bovine (M519 in chicken SV2B).

In this study, we further characterized the protein–protein interaction of BoNT/A with its protein receptors SV2A-C. The LD4 of rSV2A-C and hSV2C were expressed as glutathione-S-transferase (GST) fusion proteins and hSV2C was also expressed as His-tagged fusion protein in *Escherichia coli* (*E. coli*). GST pull down experiments showed that BoNT/A binds preferable to rSV2C-LD4 lacking the *C*-terminal TMD8, whereas addition of complex polysialo-gangliosides does not alter the interaction. H_C_A displays the highest binding affinity to hSV2C and rSV2C, but much weaker affinity to rSV2A and B. Accordingly, SV2-LD4 peptides fused to the BoNT/A *C*-terminus drastically reduced the potency of BoNT/A at the neuromuscular junction. A K_D_ of 200 nM was determined for full-length BoNT/A binding to GST-rSV2C-LD4 in pull down experiments which agrees well with the K_D_ of 550 nM determined for H_C_A to GST-rSV2C-LD4 by surface plasmon resonance (SPR) measurements. The F563 in hSV2C-LD4 significantly enhanced BoNT/A binding to hSV2C-LD4 *vs.* rSV2C-LD4 displaying a leucine instead. Circular dichroism (CD) spectroscopy revealed that the quadrilateral helix of SV2C-LD4 pre-exists in solution prior to BoNT/A binding. In comparison to the BoNT/B–Syt interaction, the recognition of SV2 by BoNT/A follows a different mechanism. In summary, these data further deepen the understanding of the BoNT/A-SV2 receptor interaction and provide a basis for developing peptidic BoNT/A inhibitors as botulism countermeasures or high affinity peptides for implementation in innovative detection systems to capture functional BoNT/A variants, e.g., from complex matrices.

## 2. Results

### 2.1. Effect of Gangliosides and SV2C Transmembrane Domain on Binding of BoNT/A to GST-rSV2C

**Figure 2:**
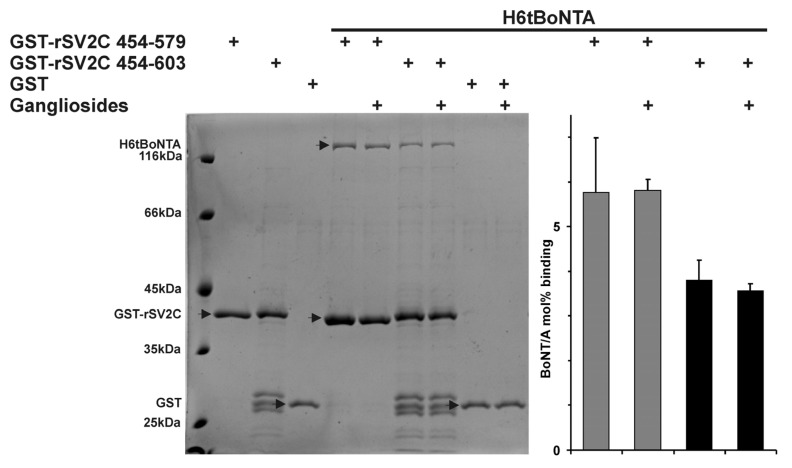
Glutathion-S-transferase (GST) pull down experiments employing GST-rSV2C 454–579 (luminal domain 4, LD4) and GST-rSV2C 454–603 (LD4 + transmembrane domain 8,TMD8) and GST (each 75 pmol), in the presence and absence of complex ganglioside mixture (125 µg) and single-chain H6tBoNTA wt (250 nM) as ligand in 100 mM Tris-HCl pH 8.0, 150 mM NaCl and 0.5% Triton X-100. Sodium dodecylsulfate polyacrylamide gel electrophoresis (SDS-PAGE) analysis of pellet samples of a representative experiment (**left**). Protein bands were stained by Coomassie blue and bands of H6tBoNTA, GST-rSV2C and GST (indicated by arrow) were densitometrically quantified. Mean ± SD of H6tBoNTA absolute binding in mol % to GST-rSV2C after subtraction of background binding to GST-GT matrix of *n* = 3 technical replicates is shown (**right**).

The neuronal uptake of BoNT/A, B, E and G occurs via a double receptor binding mechanism employing a complex polysialo-ganglioside as well as a synaptic vesicle protein receptor [11,14,30,35]. Accordingly, for BoNT/B and G it was demonstrated also *in vitro* that the presence of gangliosides synergistically increases the affinity to Syt-I and Syt-II incorporated into detergent micelles via their TMD. However, in the absence of gangliosides the affinity of Syt-I and Syt-II with TMD to BoNT/B and G decreases below that of Syt-I and Syt-II without TMD, presumably due to steric constraints at the micelle surface [11]. Similar analyses for SV2, the corresponding protein receptor for BoNT/A and E, are lacking. The LD4 of SV2 is presented by TMD7 and TMD8 into the vesicle lumen (Figure 1A). To analyze whether the BoNT/A–SV2C interaction behaves similarly, we performed GST pull down experiments employing GST-rSV2C 454–579 (LD4) and GST-rSV2C 454–603 (LD4 + TMD8) in the presence and absence of complex polysialo gangliosides and H6tBoNTA as ligand (Figure 2). Analogous to the BoNT/B–Syt interaction [11], presence of the TMD8 in GST-rSV2C 454–603 decreases the binding of H6tBoNTA by 34% compared to GST-rSV2C 454–579 and also the presence of gangliosides does not alter the binding of BoNT/A to GST-rSV2C 454–579 lacking the TMD. However, in contrast to BoNT/B–Syt interaction [11], gangliosides and SV2C plus TMD do not act synergistically on binding of BoNT/A. Here, the binding of BoNT/A to GST-rSV2C 454–603 plus gangliosides is even reduced by 38% compared to GST-rSV2C 454–579 plus gangliosides (Figure 2).

### 2.2. Binding of H_C_A to rSV2A-C Isoforms and hSV2C

**Figure 3:**
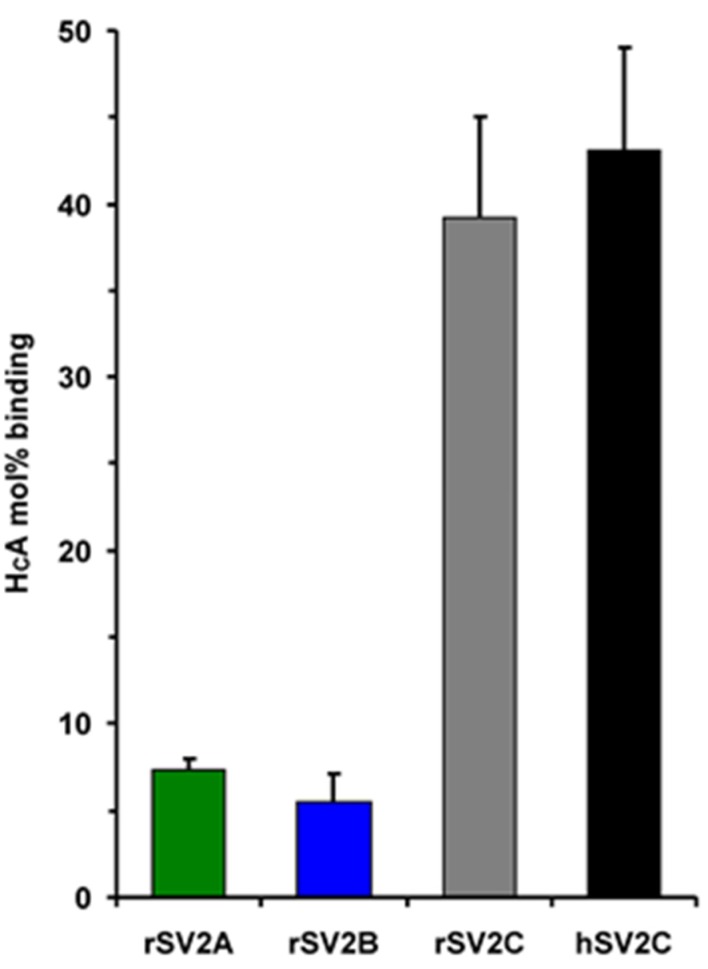
GST pull down experiments employing GST-rSV2A 468–594, GST-rSV2B 413–535, GST-rSV2C 454–579 and GST-hSV2C 455–579 (each 150 pmol) without complex gangliosides and H_C_AS wt (500 nM) as ligand in 20 mM Tris-HCl pH 7.4, 80 mM NaCl, 0.5% Triton X-100. Mean ± SD of H_C_AS absolute binding in mol % to rSV2A (*n* = 3), rSV2B (*n* = 3), rSV2C (*n* = 14) and hSV2C (*n* = 9) after subtraction of background binding to GST-GT matrix is shown.

Full-length rSV2C displays 62.3% and 59.7% AA sequence identity with rSV2A and rSV2B, respectively, whereas its LD4 (AA 454–579) is only 47.6% and 44.0% identical to rSV2A 468–594 and rSV2B 411–535, respectively (Figure 1A). The latter two LD4s are 54.4% identical to each other. Overall, only 44 of 125 residues in LD4 are identical in all three isoforms. Such diversity was suspected to influence the recognition by BoNT/A. Using GST-rSV2A 468–594, GST-rSV2B 413–535, GST-rSV2C 454–579 and GST-hSV2C 455–579 in pull down experiments, we determined the relative binding strength of the isolated cell binding domain H_C_A (Figure 3). H_C_A displayed with 5.5 ± 1.6 mol % the lowest binding to rSV2B closely followed with 7.4 ± 0.7 mol % to rSV2A. rSV2C binds 39.2 ± 5.9 mol % of H_C_A which is approximately 5- and 7-fold more than rSV2A and rSV2B, respectively. Since eleven residues of rSV2C LD4 differ in hSV2C, the latter was also analyzed for binding to H_C_A. GST-hSV2C 455–579 displayed a 10% increased binding compared to GST-rSV2C 454–579 (Figure 3), however, an unpaired *t*-test revealed a *p*-value of 0.1319 which indicates that for 500 nM H_C_A ligand the mean of GST-hSV2C 455–579 is not significantly higher than of GST-rSV2C 454–579.

### 2.3. F563L Mutation Decreases Binding of hSV2C to BoNT/A

**Figure 4:**
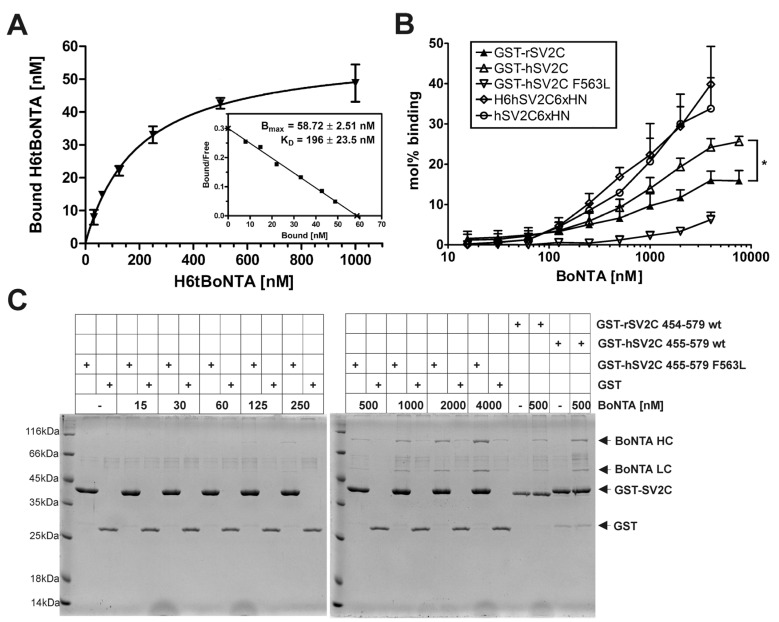
(**A**) Determination of binding constant *K*_D_ of single chain H6tBoNTA to rSV2C 454–579: GST-rSV2C 454–579 (75 pmol) was immobilized and 31 nM–1 µM single-chain H6tBoNTA in 100 mM Tris-HCl pH 7.4, 150 mM NaCl, 0.5% Triton X-100 were added as ligand (*n* = 2). Bound H6tBoNTA was quantified by SDS-PAGE and densitometry upon Coomassie blue staining. (**B**) Comparison of SV2C-LD4 fused to different affinity tags in pull down experiments. GST-rSV2C 454–579, GST-hSV2C 455–579, GST-hSV2C 455–579 F563L, H6hSV2C6xHN and hSV2C6xHN (each 75 pmol) were immobilized and tag-free di-chain BoNTA in PBS, 0.5% Triton X-100, pH 7.4 was added in ten different concentrations (15–7600 nM) as ligand (*n* = 3); (**C**) Representative SDS-PAGE analysis of a GST pull down experiment employing immobilized GST-hSV2C 455–579 F563L and tag-free di-chain BoNTA (15–4000 nM).

The binding constant K_D_ of full-length BoNT/A to rSV2C has not yet been determined. For that purpose, we performed a pull down assay with immobilized GST-rSV2C 454–579 (75 pmol) and increasing concentrations of single chain H6tBoNTA (31 nM–1 µM). Fitting a function to the saturation binding curve as well as a Scatchard plot analysis revealed a K_D_ ≈ 200 nM for H6tBoNTA (Figure 4A), which agrees well with the K_D_ of 260 nM determined for H_C_A binding to hSV2C 456–574 by fluorescence anisotropy experiments [29].

To verify whether the binding of full-length BoNT/A to hSV2C differs compared to rSV2C, tag-free fully activated BoNT/A [36] was tested as ligand in varying concentrations (15–7600 nM) in pull down assays (Figure 4B). Indeed, the affinity of BoNT/A towards hSV2C LD4 is significantly higher (*p* = 0.036) than to rSV2C LD4. As indicated in Figure 1A, four of the ten SV2C residues displaying prominent side chain interactions with BoNT/A differ between rat and human SV2C. Non-conservative substitutions are found at positions 558 (rat: Q/human: K) and 563 (rat: L/human: F). F563 of hSV2C has been reported to mediate an important cation–π interaction with R1156 of BoNT/A [29] which cannot be established by the aliphatic leucine present in rSV2C. This raised the question of whether F563 exclusively caused the difference in affinity observed between rat and human SV2C (Figure 4B). To probe its role we generated the mutant GST-rSV2C 455–579 F563L and compared its binding to BoNT/A with the two wild-type GST-SV2C constructs (Figure 4B/C). As anticipated, the binding of BoNT/A to GST-hSV2C 455–579 F563L is clearly decreased compared to its wild-type. Unexpectedly, the binding even falls below the one observed towards GST-rSV2C 454–579, suggesting that the other three residues differing might compensate the loss of the cation–π interaction between F563 and R1156 of BoNT/A.

The SV2-LD4 is presented by TMD7 and TMD8 to the SV surface. To mimic such presentation, we fused two short peptides able to bind to IMAC matrix containing Ni^2+^ or Co^2+^ to the *N*- and *C*-termini of hSV2C-LD4, respectively, yielding H6hSV2C6xHN and as a control hSV2C6xHN comprising only the *C*-terminal 6xHN-tag. Interestingly, placement of the small affinity tag to the *C*-terminus mimicking the presentation of the LD4 via its TMD8 increased binding of BoNT/A to hSV2C6xHN compared to GST-hSV2C comprising a much larger *N*-terminal tag and a freely floating SV2C *C*-terminus. However, addition of the H6-tag to the *N*-terminus and hence presenting SV2C-LD4 fixed on both termini like in full-length SV2C on the SV surface did not alter the binding to BoNT/A (Figure 4B).

### 2.4. Binding Affinity and Kinetics of H_C_A towards Immobilized GST-hSV2C 454–579

**Figure 5:**
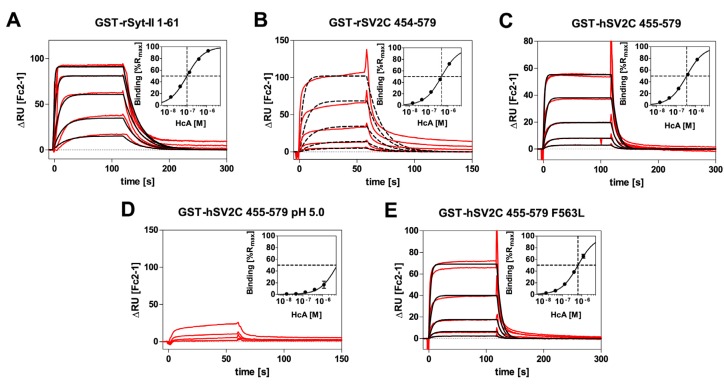
SPR-sensorgrams of the interaction of recombinant H_C_B with: GST–rSyt-II 1–61 (**A**); recombinant H_C_A with GST-rSV2C 454–579 (**B**); GST-hSV2C 455–579 (**C**); GST-hSV2C 455–579 at pH 5.0 (**D**); or GST-hSV2C 455–579 F563L (**E**) (Inserts: Fits for determination of steady-state affinity). The measured double referenced binding responses (ΔRU; red lines) are overlaid with fits of a 1:1 Langmuir interaction (black lines). For measurements of GST-rSV2C at pH 7.3 (**B**) and GST-hSV2C at pH 5.0 (**D**), kinetic evaluation was omitted due to heterogeneity in the binding curves ((**B**) dashed lines: fit for 1:1 Langmuir interaction shows clear deviations) or too low binding responses ((**D**) no fit shown). H_C_A and H_C_B were injected in three-fold dilution series starting from 1200 nM down to 14.8 nM. The highest H_C_ concentration was injected in duplicates except for GST-rSV2C 454–579 and GST-hSV2C at pH 5.0.

To further support our findings, we determined the binding kinetics and affinity of H_C_A towards immobilized GST-rSV2C 454–579, GST-hSV2C 455–579 and GST-hSV2C 455–579 F563L mutant, respectively, in comparison to the established interaction of H_C_B with GST–Syt-II 1–61 by SPR measurements (Figure 5 and Table 1). For this purpose, polyclonal rabbit anti-GST antibodies were covalently coupled to the sensor to specifically capture the GST-SV2C fusion proteins and GST as control. Such an experimental approach allows for uniform presentation of the interacting peptide with maximum degree of freedom. The measured binding responses (ΔRU; red lines) were overlaid with fits of a 1:1 Langmuir interaction (black lines; Figure 5) to yield association rate *k*_a_ and dissociation rate *k*_d_ to calculate *K*_D Kinetics_ (Table 1). As a test for consistency, *K*_D Steady-state_ values were additionally determined by fitting a steady-state affinity model to ΔRU values at equilibrium (Figure 5 and Table 1). For measurements of GST-rSV2C at pH 7.3 (B) and GST-hSV2C at pH 5.0 (D), kinetic evaluation had to be omitted due to heterogeneity in the binding curves or weak binding responses. E.g., rSV2C showed a much higher tendency to aggregate during experimental analysis, presumably by exposing hydrophobic amino acid patches which hampered the measurements due to incomplete regeneration despite inclusion of an additional regeneration step with 10 mM NaOH. Additionally, some degree of heterogeneity was observed for the interaction during SPR-measurement. A possible explanation could be that the β-sheet tertiary structure is less stable in rat than in human SV2C, making rSV2C more prone to aggregation due to exposed hydrophobic interactions. However, for rSV2C a reliable *K*_D Steady-state_ value could also be determined.

Overall, the observed interactions were characterized by rapid binding and fast dissociation and calculated *K*_D Kinetics_ were consistent with *K*_D Steady-state_ values. rSyt-II displayed a higher affinity to H_C_B than hSV2C to H_C_A due to a three-fold slower dissociation rate. In line with results of pull down assays (Figure 3), hSV2C showed slightly higher binding affinity to H_C_A than rSV2C albeit not statistically significant (*n* = 2; *t*-test: *p* = 0.16). Lowering the pH to values being measured in acidifying small synaptic vesicles [37] the *K*_D_ increases at least an order of magnitude. The lowered affinity of H_C_A towards the F563L mutant observed in the pull down assay could be confirmed by the SPR measurements. The affinity of H_C_A towards the GST-hSV2C F563L mutant was statistically significantly (*n* = 2; *t*-test: *p* = 0.0143) reduced by a factor of 1.8 as compared to GST-hSV2C wild-type. However, the effect was not as pronounced as in the pull down assay although one has to keep in mind that for SPR measurements H_C_A instead of full-length BoNT/A was used.

### 2.5. Intramolecular Blockade of SV2 Binding Site in BoNT/A

**Figure A1:**
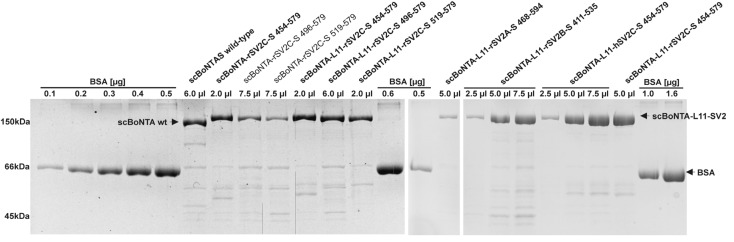
SDS-PAGE analysis of single chain scBoNTA–SV2 fusion proteins in comparison to scBoNTA wild-type. Full-length scBoNTA was fused *C*-terminally to an 11-mer linker peptide (L11) and to one of rSV2A 468–594, rSV2B 413–535, hSV2C 455–579 or a different rSV2C peptide (rSV2C 454–579 with and without L11). The potency of those constructs in bold is displayed in Figure 6.

Analogous to an earlier approach in which the Syt-II peptide fused to BoNT/B intramolecularly occupied its own binding site thereby reducing the BoNT/B potency >10-fold [32], various SV2 peptides including an 11-mer linker (L11) were fused to the *C*-terminus of BoNT/A to block the SV2 binding site. The BoNT/A–SV2 fusion proteins of up to 165 kDa were successfully generated (Figure A1). The biological activity of these fusion proteins was determined by the mouse phrenic nerve (MPN) hemidiaphragm assay [36,38].

**Figure 6:**
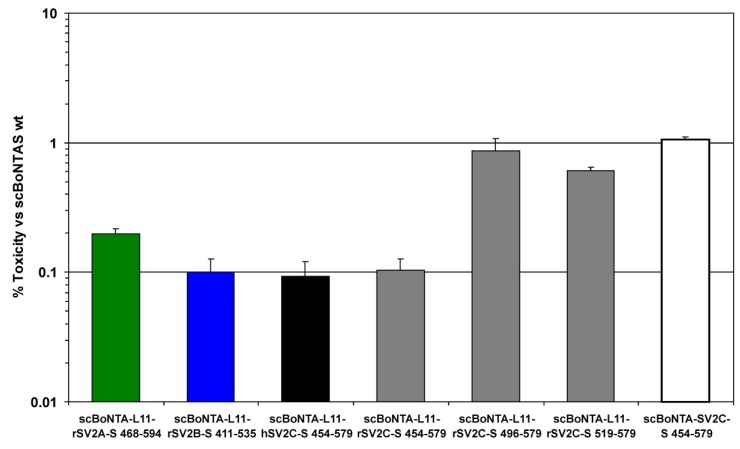
The potency of full-length BoNT/A *C*-terminally fused to an 11-mer linker peptide (L11) and to on of rSV2A 468–594, rSV2B 413–535, hSV2C 454–579 or different rSV2C peptides (rSV2C 454–579 also without L11) was determined in the MPN hemidiaphragm assay and compared to scBoNTAS wild-type. Shown is the mean ± SD of *n* = 3–5 technical replicates.

Fusion of the complete rSV2C-LD4 reduced the biological activity of BoNT/A 100-fold (Figure 6). A further 10-fold inhibition was observed upon introduction of an 11-mer linker (L11) between BoNT/A and rSV2C 454–579 supposedly providing more flexibility to the SV2C peptide to orient correctly on the BoNT/A surface. In contrast, *N*-terminal truncations of the LD4 beyond residue 473 in the presence of the linker L11 were as potent as the BoNTA-rSV2C 454–579 lacking L11. These truncations shortened the flexible linker 454–472 in SV2C and abridged the quadrilateral helix *N*-terminally, which might reduce its overall stability or conformation. Replacing rSV2C-LD4 by hSV2C-LD4 yielded similar inhibition which is in accordance with the observed binding affinity of the corresponding GST fusion proteins to H_C_A (Figure 3). Analogously, the fused rSV2A-LD4 blocked BoNT/A potency less efficiently whereas rSV2B-LD4 exhibited surprisingly similar potency as rSV2C-LD4 (Figure 6).

### 2.6. Secondary Structure Analyses of Free and Bound hSV2C Peptide

**Figure 7:**
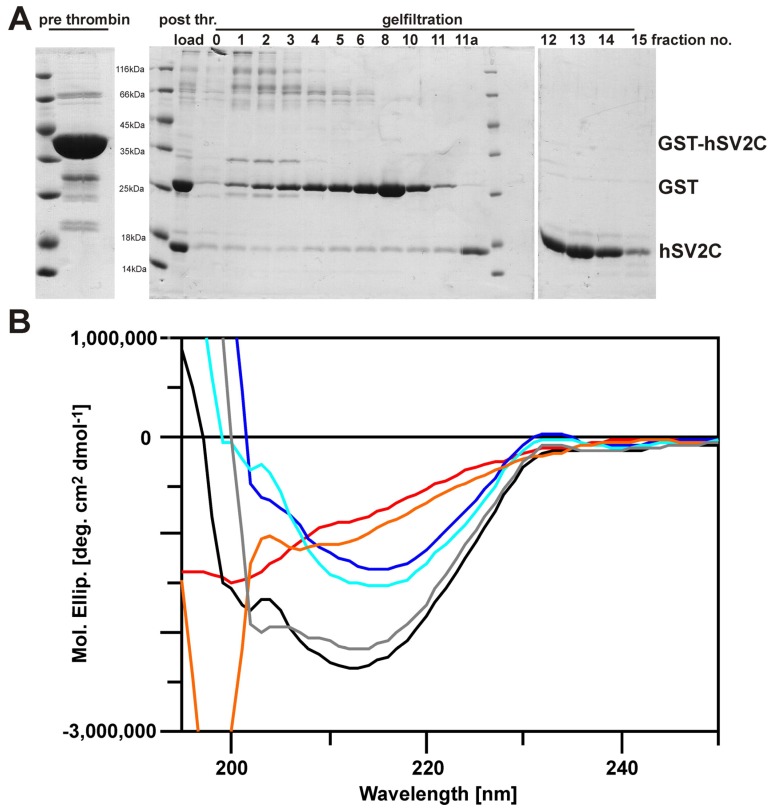
CD secondary structure analyses of free and bound hSV2C peptide. (**A**) GST-hSV2C 455–579 was quantitatively digested with thrombin and the released hSV2C peptide (15.6 kDa) was isolated by gelfiltration (fractions 11a–15). (**B**) Far-UV CD spectra of free hSV2C peptide (5 µM; red trace), free H_C_AS (8 µM, dark blue) and a 1:1 mixture of hSV2C-H_C_AS (5 µM; black). Arithmetic addition of spectra of free hSV2C (red) and free H_C_AS (dark blue) yields a spectrum (grey) similar to that of the hSV2C-H_C_AS mixture (black). Accordingly, subtraction of CD signal of either hSV2C (light blue) or H_C_AS (orange) from that of the hSV2C-H_C_AS mixture yielded spectra similar to that of the corresponding free protein.

The luminal domain of Syt-II is unstructured in solution and only upon binding to H_C_B residues E44-K60 become structured, with residues F47-I58 forming an α-helix [32]. To determine the solution structure of SV2C-LD4 we isolated the hSV2C 455–579 peptide by thrombin digest of GST-hSV2C 455–579 and subsequent gelfiltration (Figure 7A). In contrast to rSV2C 454–579, which readily precipitates upon release from GST, the hSV2C 455–579 peptide is soluble, monomeric and stable in physiological buffer. We performed CD measurements of free hSV2C peptide and H_C_AS, respectively, as well as of a 1:1 mixture of hSV2C peptide and H_C_AS to compare secondary structure of hSV2C in bound and free state (Figure 7B).

Isolated hSV2C displays a spectrum rich in β-sheet (Figure 7B, red trace) and typical for proteins folded as quadrilateral helices like *Mycobacterium tuberculosis* pentapeptide repeat protein (PRP) MtMfpA [39,40] or luminal Rfr32, a 167-residue PRP from diurnal cyanobacterium *Cyanothece* species 51142 [41,42,43]. Secondary structure estimation according to Reed and Reed [44] for the hSV2C peptide revealed 38% β-sheets, 4% turns and 58% random coil without any α-helix which agrees well with the solved crystal structure of bound hSV2C [29]. Addition of 8 M urea completely abolished its molar ellipticity signal. Isolated H_C_A yields a spectrum of a protein comprising mainly β-sheets and is similar to spectra previously determined for H_C_B and H_C_G [11]. Hereafter, H_C_AS and hSV2C peptide were mixed in a 1:1 stoichiometry. The immediate measurement yielded a spectrum dominated by β-sheet content (Figure 7B, black trace). A subsequent incubation for 30 min at 4 °C and a re-scan of the mixture showed an unaltered spectrum. To check whether secondary structure content, e.g., of the hSV2C peptide, was altered upon presence of H_C_AS, the spectra of free hSV2C and H_C_AS were arithmetically added (Figure 7B, grey trace). The summarized spectrum was virtually identical to that of the H_C_AS-hSV2C mixture (black race) indicating that no alteration in secondary structure content occurred in H_C_A and hSV2C, respectively. Conversely, the CD signal of free hSV2C or H_C_AS was subtracted from the spectrum of the H_C_AS-hSV2C mixture. In both cases, the resulting difference spectrum was highly similar to that measured of the respective isolated protein. In conclusion, hSV2C seems to fold already in solution as quadrilateral helix and binding to H_C_A does not induce a change in its secondary structure.

## 3. Discussion

The recognition of SV proteins as receptors by botulinum neurotoxins is a highly critical step in their intoxication pathway. Following the low affinity accumulation of BoNT by the abundant complex polysialo-gangliosides on the neuronal surface only a high affinity interaction with the SV protein provides sufficient specificity and avidity to enable efficient uptake of the BoNT into the lumen of small SV.

### 3.1. Unlike for BoNT/B–Syt-II, the Dual-Receptor Binding of BoNT/A–SV2C Cannot Be Reconstituted in Solution

Using a similar experimental setup as previously used for BoNT/B–Syt-II [11] we analyzed whether SV2C membrane integration and presence of complex polysialo-gangliosides contributes to BoNT/A affinity *in vitro*. Although we experimentally proved the double receptor recognition of BoNT/A recently [30], we observed neither a synergistic nor an additive effect on BoNT/A binding to SV2C in the presence of gangliosides. We speculate that the single TMD8 on the *C*-terminus important for micelle/membrane integration might fold back and binds to hydrophobic patches on the quadrilateral helix thereby blocking the surface used by BoNT/A. Alternatively, the TMD8 fails to orient the LD4 as required for BoNT/A to simultaneously bind SV2C and gangliosides. Obviously, the steric requirements for full-length BoNT/A to approach membrane juxtaposed SV2 LD4 are by far more demanding than for H_C_A which might explain the observation that isolated H_C_A displays increased binding to GST-rSV2C 454–603 in the presence of gangliosides compared to GST-rSV2C 454–579 lacking TMD8 [23]. Hence, the dual-receptor binding of BoNT/A–SV2C cannot be reconstituted in solution as for BoNT/B–Syt-II. Furthermore, mimicking the membrane presentation of SV2C-LD4 by fixing the hSV2C peptide via two affinity peptides to an IMAC matrix did not alter the binding of BoNT/A. Here, a simultaneous binding also to gangliosides could not be analyzed due to the lack of TMD8 in the H6hSV2C6xHN and hSV2C6xHN constructs.

### 3.2. Sequence Diversity in the Interacting Residues and Destabilization of Quadrilateral Helix Might Cause Lower Binding of BoNT/A to SV2A and SV2B as Compared to SV2C

The SV2A-C isoforms display a high homology and share ~60% AA sequence identity, but only 44 of 125 residues in LD4 are identical in all three isoforms. One can expect that this moderate homology in LD4 will influence physiological function and more importantly, the recognition by BoNT/A and E. Indeed, BoNT/E is only able to interact with SV2A and SV2B but not SV2C [24,35] which is most likely due to differences in AA sequence. Here, we demonstrate that rSV2A and rSV2B bind H_C_A 5–7-fold less than rSV2C, but determination of a binding constant *K*_D_ by pull down as well as SPR assays for the interaction of BoNT/A and H_C_A *vs.* SV2A and SV2B, respectively, failed because of too low affinity. A similar qualitative order of binding affinity was previously observed [22]. Despite the major backbone–backbone interactions visible in the hSV2C-H_C_A complex, ten side chain residues mediate sequence specific interactions. Of these ten residues eight are dissimilar in rSV2A and nine in rSV2B while four of them also differ between rSV2A and rSV2B. Such diversity partially explains the low affinity binding observed for BoNT/A to SV2A and SV2B and the absence of BoNT/E binding to SV2C. Analysis of the phenylalanine pentapeptide repeats causing the quadrilateral helix folding of LD4 revealed two sites each in SV2A and SV2B with a mispositioned or even lacking phenylalanine, which might impair stability or correct folding of the quadrilateral helix. Repositioning and/or replenishing the lacking phenylalanine led to increased protein solubility of the SV2A and SV2B mutants as well as increased their binding to BoNT/A [45]. Hence, differences in residues of the pentapeptide repeat folding the quadrilateral helix as well as of those ten residues interacting via their side chains with BoNT are supposed to be responsible for the low level binding of SV2A and SV2B to BoNT/A.

### 3.3. On a Species Level, Beneficial Mutations Seem to Compensate Loss of Binding Strength Caused by Deteriorating Mutations

The differences in affinity observed for hSV2C *vs.* rSV2C must be ascribed to the four residues in LD4 interacting with BoNT/A, but differing between human and rat SV2C. Of the two residues with non-conservative substitutions (Q558K, L563F), F563 of hSV2C has been reported to mediate an important cation–π-stacking interaction with R1156 of BoNT/A [29], which cannot be established by the aliphatic L563 in rSV2C. Indeed, mutant hSV2VC F563L displays clearly reduced affinity in pull down assays (Figure 4B) and moderately reduced affinity in SPR analyses (Table 1) to BoNT/A or H_C_A, respectively. Interestingly, aromatic residues at position 563 are only present in human, chimpanzee, chicken, dog, zebrafish and pufferfish SV2C, but a correlation with BoNT/A potency in those species comprising a non-aromatic AA in SV2C has not been exhibited yet.

### 3.4. SV2 Peptides Fused to BoNT/A Display a Higher Inhibitory Potency as Syt-II Peptides towards BoNT/B

Complementary to the affinity determined by pull down assays, an intramolecular inhibition of protein receptor binding was conducted to evaluate the binding strength of SV2 peptides to BoNT/A. Previously, a BoNT/B–Syt-II fusion protein displayed >10-fold reduction in potency due to autoinhibition [32]. Fusing rSV2C directly to the *C*-terminus of BoNT/A decreased its potency even 100-fold. This 10-fold higher effect may partially be attributed to the different location of Syt and SV2 binding sites in H_C_B and H_C_A, respectively. The SV2 site comprises *inter alia* the last five BoNT/A residues and fusion of any larger peptide might disturb SV2 binding. However, insertion of a 11-mer linker between the *C*-terminus of BoNT/A and the SV2C provides more flexibility of the latter to orient into its binding site and resulted in an additional 10-fold reduction of potency. Accordingly, *N*-terminal truncation of the rSV2C and replacement by rSV2A reversed potency indicating specific intramolecular inhibition of BoNT/A by SV2 peptides according to their binding site affinity.

### 3.5. The Interaction between HcA and SV2C is Highly Transient, Characterized by Rapid Binding and Unbinding

**Figure A2:**
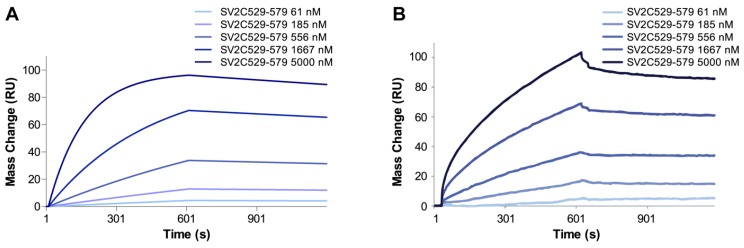
Comparison of simulated and reported SPR-sensorgrams for the interaction of immobilized H_C_A with a synthetic SV2C peptide (AA 529–579) as reported by Jacky *et al.* [46]. (**A**) Using the BIAsimulation software 2.1 (GE Healthcare), the reported kinetic binding data (*K*_D_ = 96.2 nM, *k*_a_ = 1.29 × 10^3^ M^−1^s^−1^, *k*_d_ = 1.24 × 10^−4^ s^−1^) was used to simulate the expected binding response at the indicated SV2C concentrations (61 nM to 5000 nM) assuming a maximum binding response R_max_ of 100 RU. (**B**) For comparison, the measured sensorgram was taken from Figure 3A of Jacky *et al.* [46]. Here, a mandatory overlay for the binding response of the 1:1 Langmuir interaction was not shown, so a direct appraisal of the quality of the fit was impossible. For concentrations up to 1667 nM, an acceptable agreement between the simulated and reported curves can be seen. Clear deviations are observed especially for the highest SV2C-peptide concentration tested (5000 nM) between the simulation and the measured curves. Given the reported kinetic binding data, the binding responses should begin reaching equilibrium as can be seen by a clear flattening of the binding curve for the simulation (**A**). However, the measured binding response at 5000 nM SV2C-peptide is not reaching equilibrium (**B**) indicating that the reported kinetic binding data might be inaccurate.

First SPR data on H_C_B–rSyt-II interaction displayed a fast association rate *k*_a_ despite preceding folding of the Syt-LD α-helix and an intermediate dissociation rate *k*_d_ yielding a binding constant of 75 nM which agrees well with the *K*_D_ of 34 nM [32] and 140 nM [14] determined by isothermal titration calorimetry (ITC). Although the SV2C quadrilateral helix pre-exists, H_C_A binds to hSV2C as quick as H_C_B to Syt-II, but its dissociation rate is 3-fold faster presumably due to the weaker backbone–backbone interactions. Therefore, binding constants of 500 nM for GST-rSV2C-H_C_A in SPR measurements and 200 nM for GST-rSV2C-BoNT/A in pull down assay were calculated. Hence different methods using different ligands like H_C_A or full-length BoNT/A (which could not be measured in SPR for safety reasons) largely arrive at the same overall binding affinity. The slightly lower *K*_D_ of ~400 nM for the interaction between GST-hSV2C 454–579 wild-type and H_C_A in SPR is in good agreement with previous affinity data of 260 nM determined for hSV2C 456–574 peptide by fluorescence anisotropy experiments [29] taking into account that different SV2C constructs and methods were used for measurements. However, the binding kinetics determined in this work are in strong contrast to binding kinetics of H_C_A towards a hSV2C 529–579 peptide measured in another work [46]. While the overall affinity of hSV2C 529–579 was only moderately stronger (105 nM), the association rate constant (*k*_a_ = 2.3 × 10^3^ M^−1^s^−1^) and the dissociation rate constant (*k*_d_ = 2.5 × 10^−4^ s^−1^) were significantly slower by a factor of 135 and 480, respectively. Jacky *et al.* [46] covalently immobilized H_C_A by amine coupling to a CM5 sensor chip yielding a random presentation. In contrast, we captured the GST-SV2C peptides by anti-GST antibodies, where the SV2C is uniformly oriented to the solvent. Furthermore, Jacky *et al.* [46] injected the synthetic hSV2C 529–579 peptide dissolved in DMSO over the sensor surface. The requirement of using DMSO as solvent indicates that the hydrophobic core of the quadrilateral β-sheet helix [29] might be solvent exposed due to misfolding [47] which renders the molecule rather “sticky”. This would explain the pronounced reduction in both association and dissociation rate constants, hereby falsifying the interaction. On the contrary, we used a directed capture approach for capturing GST-SV2C 454–579 and injected H_C_A in physiological buffer over the sensor surface, hereby mirroring the natural presentation of the SV2C receptor on the surface of the presynaptic membrane [22,23] without potential disturbances by covalent immobilization of H_C_A and denaturation of hSV2C by solvent [47]. Finally, the mandatory overlay of the curve fitting employing the 1:1 kinetic-binding model (A + B ↔ AB) with the raw data is missing in Jacky *et al.* [46] and disables the reader to evaluate the quality of curve fitting. Simulating the H_C_A-hSV2C 529–579 interaction by the BIA simulation software 2.1 employing the 1:1 kinetic-binding model (A + B ↔ AB), the above cited *k*_a_, *k*_d_ and *K*_D_ and five hSV2C peptide concentrations surprisingly yields curves (Figure A2) which do not fit with the sensorgrams given in Figure 3A of Jacky *et al.* [46] and questions the validity of their SPR study. Taking into account this finding and considering the influence of electrostatic charge interactions on the binding between H_C_A and hSV2C [29], which are known to enhance association rate constants [48], let us conclude that the more rapid association and dissociation rate constants reported here describe the BoNT/A–SV2C interaction more accurately.

### 3.6. A Preformed Structure in Solution and pH-Dependent Binding Distinguish SV2C and Syt-II

The increase of the *K*_D_ at pH 5.0 which reflects the situation of BoNT/A in acidifying small SV is in contrast to the BoNT/B–Syt-II interaction which is pH-independent [32]. Due to the differently localized protein receptor binding sites it might be necessary for BoNT/A to dissociate from SV2C to be able to reorient prior to membrane insertion as prerequisite for pore formation and LC translocation. Along that line, it was shown that H_C_ mediates pH-dependent translocation [49].

In contrast to Syt-I and -II which change their unstructured luminal domain upon BoNT binding into an α‑helix which exclusively forms side chain interactions with BoNT/B and G [50], our CD data suggests that isolated SV2 LD4 exists as preformed quadrilateral β-sheet right handed helix as observed in the X-ray structure [29]. Fixation of this stable helix by two transmembrane anchors to the vesicle lumen then allows for the establishment of backbone–backbone interactions within a small interface of H_C_A at a site distinct to the Syt site in BoNT/B and G.

## 4. Conclusions

Here, we have further characterized the BoNT/A–SV2 protein–protein interaction. BoNT/A displays the highest binding affinity to hSV2C and rSV2C, but much weaker affinity to rSV2A and B. It binds preferable to rSV2C-LD4 lacking the *C*-terminal TMD8 independent of complex polysialo-gangliosides which is in sharp contrast to the BoNT/B–Syt-II receptor interaction. Accordingly, SV2-LD4 peptides fused to the BoNT/A *C*-terminus drastically reduced the potency of BoNT/A at the neuromuscular junction. Fast association and dissociation of BoNT/A to SV2C-LD4 pre-existing as quadrilateral helix yields binding constants of 200–500 nM. The F563 in hSV2C-LD4 enhances BoNT/A binding to hSV2C-LD4 *vs.* rSV2C-LD4 displaying a leucine instead, whereas acidic pH measured in SV drastically weakens the BoNT/A–SV2C interaction. In conclusion, the mechanism and way of receptor recognition of BoNT/A clearly diverges from BoNT/B which might be attributable to the different location of the protein receptor binding sites in BoNT/A and B. Our data further deepen the molecular understanding of the BoNT/A–SV2 receptor interaction and supports the generation of novel SV2-based peptides as inhibitors to counteract deliberate release of BoNT/A and E and as high affinity agents in innovative detection systems to capture functional BoNT/A variants e.g., from complex matrices to prevent and diagnose botulism.

## 5. Materials and Methods

### 5.1. Plasmid Construction

Plasmids encoding the H_C_-fragment of BoNT/A fused to a *C*-terminal Streptag (H_C_AS), single chain full-length BoNT/A equipped with an *N*-terminal, thrombin-cleavable His6tag (H6tBoNTA), full-length BoNT/A with thrombin-cleavable tags (BoNTA) as well as GST fusion proteins of rSV2A 468–594, rSV2B 413–535, rSV2C 454–579 and rSV2C 454–603 were described previously [9,23,30,36].

The plasmid pGEXhSV2C 455–579 encoding GST-hSV2C 455–579 was generated by cloning the synthetic *E. coli* codon usage optimized DNA sequence encoding hSV2C 455–579 (Geneart, Regensburg, Germany) into a pGEX-4T3 vector cut with BamH I/Xma I. The mutation F563L was inserted into pGEXhSV2C 455–579 by PCR applying the GeneTailor™ site-directed mutagenesis system (Life Technologies, Darmstadt, Germany) and suitable primers (Eurofins, Ebersberg, Germany). pGEXhSV2C-6xHN 455–579 was generated by inserting a DNA fragment encoding a 6xHN tag into pGEXhSV2C 455–579 via Xma I/Xho I sites. The plasmid encoding pGEXH6hSV2C-6xHN 455–579 was generated from pGEXhSV2C-6xHN 455–579 by inserting a DNA-fragment encoding a H6-tag employing the 5′ BamH I site.

Expression vectors encoding variants of BoNTA-SV2-S fusion proteins were generated by opening pBoNTAS wt at the bont/A 3′ end using the unique Sma I site [9] and inserting Sma I digested PCR products which contain an additional EcoR V site at the 5′ end and were amplified from pGEXrSV2A 468–594, pGEXrSV2B 413–535, pGEXrSV2C 454–579 and pGEXhSV2C 455–579. The expression vectors encoding the BoNTA-L11-SV2-S fusion proteins were generated by linearizing the corresponding pBoNTA-SV2-S plasmids at the unique EcoR V site and inserting a synthetic blunt end DNA fragment encoding the linker L11 (GGSGSSGSSGA).

Nucleotide sequences of all newly generated constructs were verified by DNA sequencing (GATC Biotech, Konstanz, Germany).

### 5.2. Expression and Purification of Recombinant Proteins

The generation of H_C_AS, scBoNTAS, single-chain H6tBoNTA and activated full-length BoNTA were described previously [9,30,36]. Expression and purification of the fusion proteins scBoNTA-rSV2C-S, scBoNTA-L11-rSV2C-S and variants thereof was conducted analogously to that of scBoNTAS wild-type under biosafety level 2 containment (project number GAA A/Z 40654/3/123) [9].

GST fusion proteins (GST-rSV2A 468–594, GST-rSV2B 413–535, GST-rSV2C 454–579, GST-rSV2C 454–603 were purified as described earlier [23]. The novel GST-hSV2C 455–579, GST-hSV2C 455–579 F563L, GST-hSV2C-6xHN 455–579 and GST-H6hSV2C-6xHN 455–579 were produced analogously. GST fusion proteins eluted by glutathione were dialyzed against PBS, pH 7.4, two times with, and two times without β-mercaptoethanol. GST-hSV2C-6xHN 455–579 and GST-H6hSV2C-6xHN 455–579 stayed bound to glutathione-sepharose-4B matrix (GE Healthcare, Freiburg, Germany) while GST-hSV2C 455–579 was eluted and were treated for 16 h shaking at room temperature with 0.01 U bovine thrombin (Sigma-Aldrich Chemie GmbH, Steinheim, Germany) per µg protein in 0.1 M Tris-HCl pH 8.0, 150 mM NaCl supplemented with 1 mM CaCl_2_ to cleave off the SV2C peptide from the GST-tag. The released (H6)hSV2C6xHN-peptides were isolated from the supernatant using Co^2+^-Talon matrix (Takara Bio Europe S.A.S., Saint-Germain-en-Laye, France) and eluted with 50 mM Tris-HCl, pH 8.0, 150 mM NaCl, 250 mM imidazole. The released hSV2C-peptide was purified from the supernatant by gelfiltration in PBS, pH 7.4. Desired fractions were pooled, dialyzed against PBS pH 7.4, frozen in liquid nitrogen and kept at −70 °C.

Protein concentrations were determined subsequent to SDS-PAGE and Coomassie blue staining by using a LAS-3000 imaging system (FUJIFILM Europe GmbH, Düsseldorf, Germany), the AIDA 3.51 software (Raytest, Straubenhardt, Germany) and BSA (100–1600 ng) as reference protein.

### 5.3. Pull Down Assay

The GST pull down assays were similarly performed as previously described [30] with the addition of 125 µg ganglioside mixture (Matreya, State College, PA, USA) in selected experiments as indicated.

Briefly, GST, GST fusion proteins and 6xHN-tagged fusion proteins (75 or 150 pmol each) were immobilized to 10 µL glutathione-sepharose-4B matrix (Qiagen, Hilden, Germany) or 5 µL Co^2+^-Talon matrix (Takara Bio Europe S.A.S., Saint-Germain-en-Laye, France), respectively, and subsequently incubated for 2 h at 4 °C with H_C_AS, single chain H6tBoNTA or di-chain BoNTA in a total volume of 100–200 µL in binding buffer as stated in the respective figure legends. Beads were collected by centrifugation and washed two times each with the corresponding binding buffer. Washed pellet fractions were incubated at 37 °C for 20 min in SDS sample buffer and analyzed by 10% SDS-PAGE. Protein bands were detected by Coomassie blue staining and subsequently quantified by densitometry using the software TINA (version 2.09f, Raytest, Straubenhardt, Germany). Unspecific binding of ligand to immobilized GST or free Co^2+^-Talon matrix was subtracted from the specific binding signal of H_C_A or BoNTA to SV2. *K*_D_ values and statistics were calculated with GraphPadPrism (version 4.03 January 2005, GraphPad Software Inc., La Jolla, CA, USA).

### 5.4. Surface Plasmon Resonance Measurements (SPR)

SPR measurements to determine binding kinetics and/or affinity of recombinant H_C_A-fragments towards GST-hSV2C 455–579 and GST-rSV2C 454–579 were performed using a Biacore X100 unit (GE Healthcare, Freiburg, Germany) at 25 °C. PBS (pH 7.3) or 51.4 mM Na_2_HPO_4_, 24.3 mM Citrate, 150 mM NaCl (pH 5.0) supplemented with 0.5% Triton X-100 were used as running buffer. For GST-rSyt-II 1–61, HBS-EP+ (GE Healthcare, Freiburg, Germany) was used as running buffer. Recombinant GST-fusion proteins were immobilized employing the GST capture kit (GE Healthcare, Freiburg, Germany) according to the manufacturer’s recommendations. Briefly a CM5 sensor chip was covalently coupled with rabbit anti-GST capture antibodies. For each measurement, GST-hSV2C 455–579 (wt or F563L) or GST-rSV2C 454–579 and, as a negative control, recombinant GST (both at 5 µg/mL) were captured for 30 s at a flow rate of 5 µL/min on flow cells 2 or 1, respectively, leading to immobilization levels of approximately 200 resonance units (RUs).

For kinetic measurements, three-fold dilution series of H_C_A or HcB (1200, 400, 133, 44, 14.8 nM) were injected over both flow cells for 60 or 120 s at a flow rate of 30 µL/min. Dissociation was monitored subsequently for 120 or 300 s by running buffer injections. Between measurements, the sensor surface was regenerated by injections of 10 mM glycine-HCl (pH 2.1) for 120 s over both flow cells. Due to incomplete regeneration for GST-rSV2C 454–579 measurements and GST-hSV2C 455–579 (pH 5.0), an additional injection of 10 mM NaOH for 15 s at 10 µL/min was necessary to completely remove bound H_C_A from the flow cells. Duplicate injections of the highest H_C_A concentration as well as triplicate injections of running buffer only were included. Due to the harsh regeneration conditions necessary during GST-rSV2C 454–579 and pH 5.0 measurements, a gradual loss of immobilization levels was observed. Therefore, the duplicate measurement of the highest H_C_A concentration had to be omitted from this analysis to enable robust global fitting of binding affinities.

Kinetic association and dissociation binding rate constants (*k*_a_ and *k*_d_) and the equilibrium dissociation constant *K*_D_ were determined by fitting double referenced sensorgrams [51] to a 1:1 Langmuir interaction model with RI set to 0 and R_max_ set global using the Biacore evaluation software (v2.01). *K*_D_ values were additionally determined by fitting to a steady-state affinity model as a test for consistency between kinetic and affinity analysis of the interactions.

### 5.5. Potency of BoNT/A–SV2 Fusion Proteins at MPN Hemidiaphragm Assay

Intramolecular inhibition of BoNT/A by fusing various SV2 peptides to the *C*-terminus of scBoNTAS was determined employing the MPN assay [36,38]. In brief, the left phrenic nerve hemidiaphragm was excised from euthanized female mice of strain RjHan:NMRI (18–25 g, Janvier Labs, Genest Saint Isle, France), placed in an organ bath and equilibrated for 15 min in 4 mL of Earle’s Balanced Salt Solution, pH 7.4 gassed with 95% O_2_ and 5% CO_2_. The phrenic nerve was continuously electro-stimulated (frequency 1 Hz, 0.1 ms pulse duration, 25 mA current). Isometric contractions (resting tension ~10 mN) were recorded with a force transducer (Scaime, Annemasse, France) and the software VitroDat (Föhr Medical Instruments GmbH (FMI), Seeheim, Germany). Toxin was applied in 4 mL of Earle’s Balanced Salt Solution supplemented with 0.1% BSA in concentrations to allow 50% decay of the contraction amplitude within 50 to 150 min. The times required to decrease the amplitude by 50% (paralysis time t_½_ ≤ 180 min) for three BoNT concentrations were used to construct the calibration curves for scBoNTAS to which a power function was fitted (*y* (scBoNTAS; 0.8/3/8 pM) = 108.61*x*^−0.355^, *R*^2^ = 0.894). This power function was used to convert the paralysis times t_½_ determined for BoNTA-SV2 constructs into the corresponding scBoNTAS wt concentrations and expressed as relative biological activity of scBoNTAS wt.

### 5.6. Circular Dichroism Analysis

Circular dichroism (CD) data was collected with a Jasco J-810 spectropolarimeter (JASCO International Co. Ltd., Tokyo, Japan) in a 1 mm path length cuvette with a concentration of 5–8 µM degassed H_C_A and 5 µM hSV2C in PBS pH 7.4. Spectra were recorded at 22 °C from 195 to 250 nm with 100 nm/min, response of 1 s, standard sensitivity, bandwidth of 1 nm and five accumulations. Spectra were analyzed, processed and visualized using Spectra Manager II software (version 2.06, JASCO International Co. Ltd., Tokyo, Japan, 2007).

## Figures and Tables

**Table 1:** Kinetic binding rate constants (*k*_a_ and *k*_d_) and equilibrium dissociation constants (*K*_D_) for the interaction of H_C_B with GST-rSyt-II and H_C_A with GST-SV2C variants.

Ligand	pH	*k*_a_ [M^−1^s^−1^]	*k*_d_ [s^−1^]	*K*_D Kinetics_ [M]	*K*_D Steady State_ [M]
**GST-rSyt-II 1–61**	7.4	5.4 ± 0.3 × 10^5^	4.1 ± 0.2 × 10^−2^	7.5 ± 0.8 × 10^−8^	7.2 ± 1.2 × 10^−8^
**GST-rSV2C 454–579**	7.3	n.a.	n.a.	n.a.	5.1 ± 0.3 × 10^−7^
**GST-hSV2C 455–579**	7.3	3.1 ± 0.5 × 10^5^	1.2 ± 0.1 × 10^−1^	3.9 ± 0.5 × 10^−7^	4.0 ± 0.6 × 10^−7^
**GST-hSV2C 455–579**	5.0	n.a.	n.a.	n.a.	>1.2 × 10^−6^
**GST-hSV2C 455–579 F563L**	7.3	1.9 ± 0.1 × 10^5^	1.3 ± 0.1 × 10^−1^	6.8 ± 0.1 × 10^−7^	6.6 ± 0.3 × 10^−7^

Shown are mean ± SD of *n* = 2 technical replicates; n.a. = not applicable.

## Abbreviations

The following abbreviations are used in this manuscript:

AA: amino acid
BoNT: botulinum neurotoxin
CD: circular dichroism
GBS: ganglioside binding site
GST: glutathion-S-transferase
H: human
HC: heavy chain
H_C_: carboxyl-terminal half of HC
H_C_A, H_C_B, H_C_E: H_C_ fragment of BoNT serotype A, B and E, respectively
H_CN_ and H_CC_: 25 kDa halves of H_C_
H_N_: amino-terminal half of HC
H6: *N*-terminal 6x His tag
6xHN: (HisAsn)_6_ tag
kDa: kilo Dalton
L11: 11-mer linker peptide
LC: light chain
LD: luminal domain
MPN assay: mice phrenic nerve hemidiaphragm assay
MW: molecular weight
PRP: pentapeptide repeat protein
RU: resonance unit
S: *C*-terminal Streptag
SDS-PAGE: sodium dodecylsulfate polyacrylamide gel electrophoresis
SNAP-25: synaptosomal associated protein of 25 kDa
SNARE: soluble *N*-ethylmaleimide-sensitive factor attachment protein receptor
SPR: surface plasmon resonance
SV: synaptic vesicle
rSV2A-C: rat synaptic vesicle glycoprotein 2 isoform A, B or C
Syt: synaptotagmin
TMD: transmembrane domain
